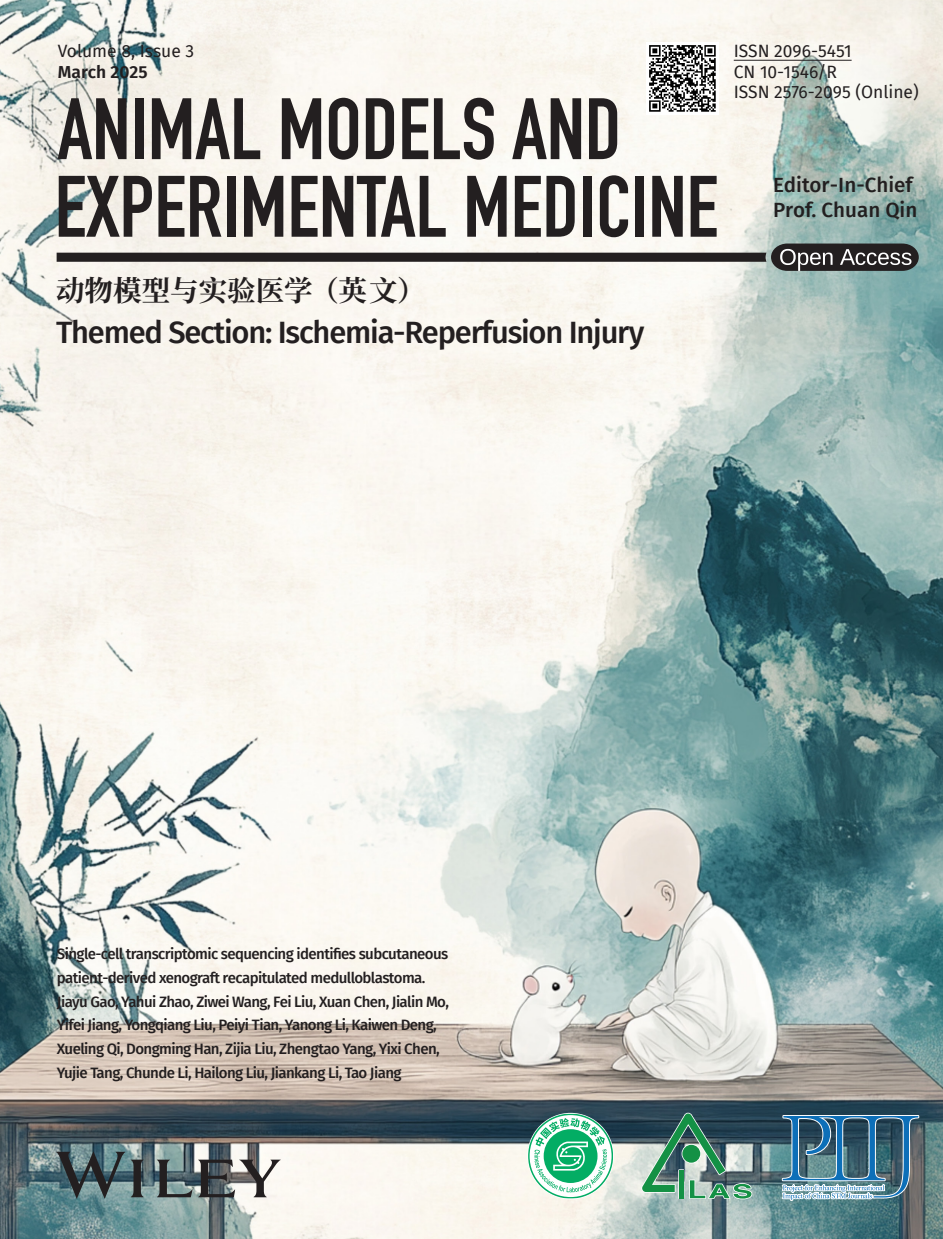# Cover Picture

**DOI:** 10.1002/ame2.12426

**Published:** 2025-03-12

## Abstract

This cover image is based on the article “Single‐cell transcriptomic sequencing identifies subcutaneous patient‐derived xenograft recapitulated medulloblastoma” (https://doi.org/10.1002/ame2.12399) reported by Jiayu Gao, Yahui Zhao, Ziwei Wang, Fei Liu, Xuan Chen, Jialin Mo, Yifei Jiang, Yongqiang Liu, Peiyi Tian, Yanong Li, Kaiwen Deng, Xueling Qi, Dongming Han, Zijia Liu, Zhengtao Yang, Yixi Chen, Yujie Tang, Chunde Li, Hailong Liu, Jiankang Li, Tao Jiang. The image mainly conveys children's gratitude for the contributions made by laboratory mice to disease research and drug screening.